# Neurotransmitters of sleep and wakefulness in flatworms

**DOI:** 10.1093/sleep/zsac053

**Published:** 2022-03-07

**Authors:** Shauni E T Omond, Matthew W Hale, John A Lesku

**Affiliations:** 1 School of Agriculture, Biomedicine and Environment, La Trobe University, Melbourne, Australia; 2 School of Psychology and Public Health, La Trobe University, Melbourne, Australia

**Keywords:** dopamine, GABA, *Girardia*, histamine, nematode, platyhelminth, pyrilamine

## Abstract

**Study Objectives:**

Sleep is a prominent behavioral and biochemical state observed in all animals studied, including platyhelminth flatworms. Investigations into the biochemical mechanisms associated with sleep—and wakefulness—are important for understanding how these states are regulated and how that regulation changed with the evolution of new types of animals. Unfortunately, beyond a handful of vertebrates, such studies on invertebrates are rare.

**Methods:**

We investigated the effect of seven neurotransmitters, and one pharmacological compound, that modulate either sleep or wakefulness in mammals, on flatworms (*Girardia tigrina*). Flatworms were exposed via ingestion and diffusion to four neurotransmitters that promote wakefulness in vertebrates (acetylcholine, dopamine, glutamate, histamine), and three that induce sleep (adenosine, GABA, serotonin) along with the H1 histamine receptor antagonist pyrilamine. Compounds were administered over concentrations spanning three to five orders of magnitude. Flatworms were then transferred to fresh water and video recorded for analysis.

**Results:**

Dopamine and histamine decreased the time spent inactive and increased distance traveled, consistent with their wake-promoting effect in vertebrates and fruit flies; pyrilamine increased restfulness and GABA showed a nonsignificant trend towards promoting restfulness in a dose-dependent manner, in agreement with their sleep-inducing effect in vertebrates, fruit flies, and *Hydra*. Similar to *Hydra*, acetylcholine, glutamate, and serotonin, but also adenosine, had no apparent effect on flatworm behavior.

**Conclusions:**

These data demonstrate the potential of neurotransmitters to regulate sleep and wakefulness in flatworms and highlight the conserved action of some neurotransmitters across species.

**Statement of Significance**

Although sleep, as a behavior, is widespread across the animal kingdom, whether the physiology and regulatory machinery associated with sleep and wakefulness, are also conserved remains unclear. We exposed flatworms (*n* = 504) to seven neurotransmitters, and one pharmacological compound, that regulate either sleep or wakefulness in mammals. The flatworm (phylum Platyhelminthes) are simple animals that lack circulatory, respiratory, and endocrine systems found in other bilaterally symmetric animals, and yet they sleep and possess a bilobal brain. We found that GABA showed a nonsignificant trend to induce sleep in flatworms, and histamine and dopamine promoted wakefulness. We conclude by casting our results into a phylogenetic framework to trace evolutionary similarities, and differences, in neurotransmitter function across distantly related types of animals.

## Introduction

Sleep and wakefulness are two behavioral and physiological states exhibited by most, and perhaps all, animals [1]. These two states are characterized by variant responses to external stimuli. For instance, awake animals detect and respond to salient environmental cues readily, whereas sleeping animals show reduced responsiveness to stimulation. Sleep is also characterized by restfulness and homeostatic regulation. These states have been extensively studied in mammals and other vertebrates [2–5], and to a lesser extent invertebrates, including arthropods [6] and mollusks [7], roundworms [8], flatworms [9], jellyfish [10], and *Hydra* [11].

In vertebrates, sleep and wake are modulated by conserved neurotransmitter systems. Neurotransmitters such as acetylcholine, dopamine, glutamate, and histamine promote wakefulness [12–14]; others, including adenosine, serotonin, and ƴ-aminobutyric acid (GABA), promote sleep [12,14]. How neurotransmitters regulate sleep and wake among invertebrates is comparatively lacking. What is known from these lesser-studied groups comes from a handful of species. The fruit fly (*Drosophila melanogaster*) shows that many of these neurotransmitters produce the same behavioral output as observed in vertebrates [15,16]. There is less similarity between the behavioral responses of vertebrates and cnidarians, the most basal group of animals with a nervous system. GABA, melatonin (a hormone that promotes sleep in diurnal animals), and pyrilamine (a sleep-promoting H1 histamine receptor antagonist) likewise induce sleep in the cnidarian, *Hydra* [10,11] Acetylcholine, glutamate, histamine, and serotonin, conversely, have no effect on *Hydra* behavior [11]. Interestingly, dopamine was found to be sleep-promoting in *Hydra*, rather than wake-promoting, as observed in all other taxa studied [11,17], demonstrating that at least some neurotransmitters are evolutionarily labile.

The relative lack of comparative data on the behavioral response of invertebrates to neurotransmitters has been the main impediment to understanding how neurotransmitter systems have changed over evolutionary time. Additional data, particularly on unstudied animal phyla, are needed to ascertain which elements of the sleep/wake regulatory machinery are evolutionarily conserved, and which are derived. To this end, we introduce the platyhelminth flatworm.

Although flatworms sleep [9], we know nothing about how neurotransmitters regulate sleep and waking states in platyhelminths. Flatworms are simple invertebrates that lack circulatory, respiratory, and endocrine systems [18]. This simplicity is useful for dissecting the effect of neurotransmitters, which in some taxa, have non-neural biological targets. Several studies on neurotransmitter systems have been conducted on free-living and parasitic flatworms, but on isolated cells or muscle fibers, or without consideration of circadian timing [19–30]. While looking at these effects at a cellular level may be helpful, whole animal behavioral assays are needed to obtain the clearest picture of the effects on the individual [11,31,32]. Here, we add new insight into the effect of seven common neurotransmitters, and one pharmacological compound, on sleep and wakefulness in flatworms.

## Methods

### Animals

Free-living flatworms (*Girardia tigrina*) were wild-caught by Southern Biological (Melbourne, Australia) and housed at La Trobe University (n = 504). There they were kept in a temperature-controlled room at 14 ± 1°C and entrained to a 12:12 light:dark cycle with lights-off at 20:00 h. Each flatworm was exposed to only one concentration of only one compound to avoid any lingering effects of a compound (see Supplementary Table S1 for a complete list of sample sizes). Flatworms were initially group-housed (approx. 50 animals per container) in glazed ceramic bowls 15 cm in diameter filled with spring water. Animals were offered hard-boiled egg yolk twice weekly for 120 min; postfeeding they were transferred to a clean bowl with fresh water. Flatworms were fasted prior to experimental recording to ensure they were motivated to eat the neurotransmitter-imbued egg yolk; several fasting durations had been piloted with 14 days yielding good intake. Flatworms, as simple ectothermic invertebrates, have low energy requirements and do not need to eat nearly as frequently as many other animals.

A custom-built activity monitoring system was used to illuminate, and video record, the testing arenas [9]. Briefly, the system consisted of two box frames with a roof and two walls. Within each box, there was a single camera (A602f; Basler AG, Ahrensburg, Germany) placed above a testing area, with dim LEDs (64 ± 2 lux) placed upon the sides of the walls to simulate the attenuation of sunlight that occurs in a pond environment in which the flatworms are found in the wild. The camera had four aluminum arms that extended to the edges of the testing area below; the end of each arm was fitted with infrared LEDs (950 nm). The infrared lights provided constant illumination to the cameras to ensure no difference in image quality between light and dark, and flatworms do not respond to infrared light [33].

### Compounds

All compounds were obtained from Sigma-Aldrich Pty Ltd (Castle Hill, Australia) and reconstituted from powder. Neurotransmitters that promote wakefulness in mammals are acetylcholine (Cat. A6625), dopamine (Cat. H8502), glutamate (Cat. G1251), and histamine (Cat. H7125); and compounds that promote sleep are adenosine (Cat. 0.1890), ɣ-aminobutyric acid (GABA, Cat. A2129), pyrilamine (Cat. P5514), and serotonin (Cat. 14927).

**Figure 1.**
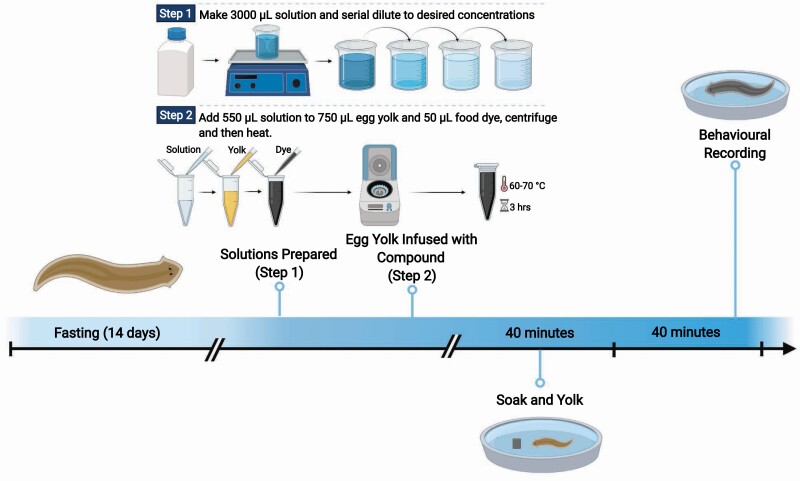
Neurotransmitter preparation (*top*) and experimental design (*bottom*): the flatworms were fasted for 14 days, and then presented with the drug-infused egg yolk, and immersed in drug-treated water, for 40 minutes prior to the experiment (“soak and yolk”). Animals were transferred to spring water for the duration of the recording (40 minutes); the initial 10 minutes were excluded from analysis owing to acclimation. Created with BioRender.com.

**Figure 2.**
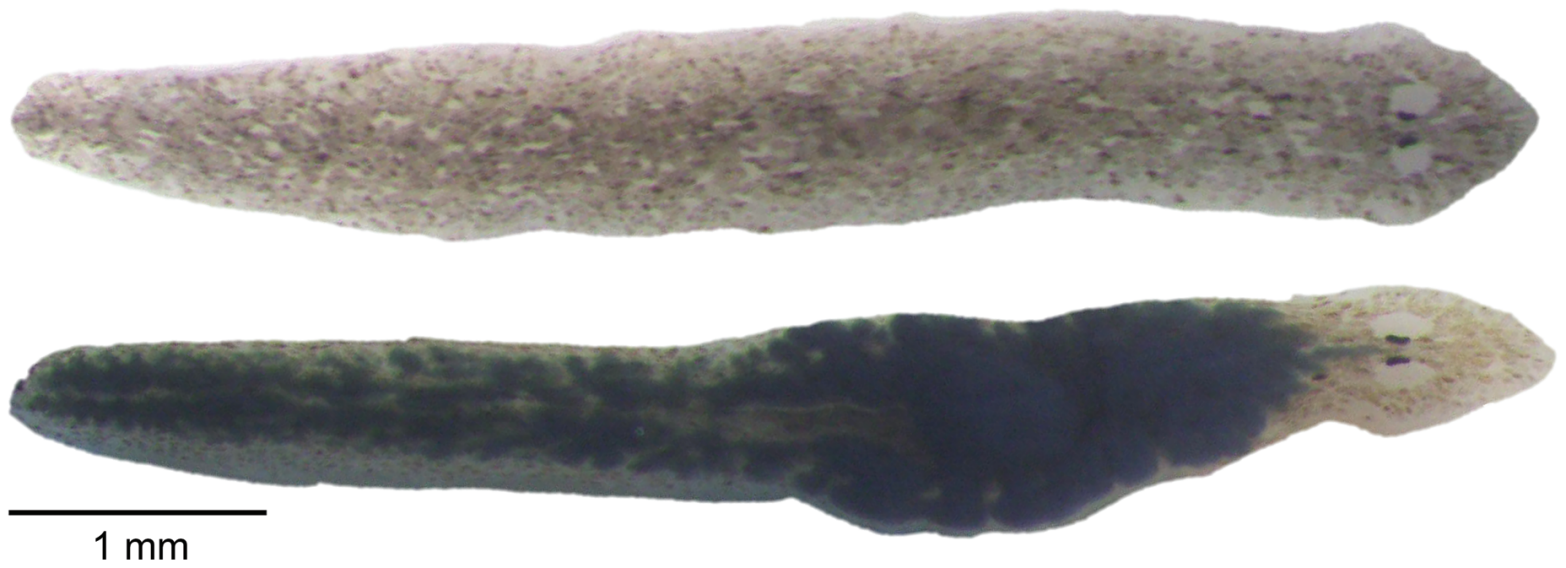
Photograph of a flatworm that has not ingested the compound-imbued egg yolk (*top*), and an animal that has consumed the black-dyed, drug-rich, egg yolk and, therefore, exhibits a visible change in color (*bottom*).

Compounds were mixed into a 3,000 μM solution and serially diluted to four concentrations (i.e., 300 μM, 30 μM, 3 μM, and 0.3 μM), which were used to infuse egg yolk with a specific compound (Figure 1). Five hundred μL of compound was added to 950 μL of egg yolk and 50 μL of food coloring; the addition of food coloring allowed us to visually determine which flatworms had eaten and then exclude those that had not (Figure 2). The resulting mixture was pipetted into Eppendorf tubes, vortexed, and heated at 60–70°C in a water bath until solid. Histamine, stored at –20°C, was made into a solution and thawed to make the egg yolk. Solutions and infused egg yolks were made fresh for each trial such that there was only one freeze-thaw cycle. The animals were exposed to the infused egg yolk for 40 minutes prior to the experiment.

The 3,000 μM stock solution was also diluted to 1,000 μM, 100 μM, 10 μM, 1 μM, and 0.1 μM, to be used as a bath for the animals immediately prior to the experiment (Figure 1). Fifteen mL of these weaker solutions were used as a bath to match the concentration of the egg yolk (mentioned above). This ensured that there was no osmotic gradient and the dosage of the compound remained constant throughout the 40 minutes. High concentrations of serotonin (1,000 µM) and pyrilamine (100 μM, 1,000 µM) produced adverse behavioral reactions and were excluded from analysis; low samples sizes excluded 1,000 µM of histamine and glutamate from analysis.

### Recording

Animals were transferred to small wells filled with 2 mL of spring water and behavior was recorded for 40 minutes (Figure 1); the first 10 minutes of the trial was excluded owing to an acute increase in movement whenever the animals are moved to the new environment. The pharmaceutical compounds that promote wakefulness in mammals (acetylcholine, dopamine, histamine) were administered to the flatworms during the first half of the day (between 08:30–11:30 h); purportedly sleep-promoting neurotransmitters (adenosine, GABA, pyrilamine, serotonin) were tested during the first half of the night (between 20:30–23:30 h). Glutamate was tested as sleep-promoting in flatworms, given ostensibly sleep-promoting effects in fruit flies [15,34]. Time frames were so chosen, because flatworms are strongly nocturnal [9], such that wake-promoting compounds were presented at the time-of-day when flatworms would otherwise be asleep. With this hypothesis-testing experimental design, we were able to determine whether each chemical similarly effected flatworm and vertebrate behavior, but only in one-half of the 24-h day.

### Analysis

Video recordings were analyzed using EthoVision XT 10 (Noldus Information Technology, Wageningen, The Netherlands) to track the amount (%) and level (distance traveled) of activity. Statistical analyses were conducted using GraphPad Prism (version 9.0 for Windows, GraphPad Software, La Jolla, USA). Based on our previous work with flatworms demonstrating that rest/activity cycles reflect sleep/wake differences [9], we used inactivity as a proxy for sleep and activity as a marker for wake.

We confirmed that data conformed to the assumptions of parametric statistical tests using a Kolmogorov-Smirnov test. When data was non-normal, we tried to achieve normality using a log_10_ and square root transformation. In the distance traveled data, acetylcholine and pyrilamine were square root transformed, and glutamate was log_10_ transformed. For the % inactive data, only acetylcholine was normal and was analyzed using a one-way ANOVA with Dunnett’s posthoc comparisons; all other neurotransmitters could not be normalized and were analyzed using a Kruskal-Wallis with Dunn’s posthoc comparisons. For the distance traveled data, we used a one-way ANOVA with Dunnett’s posthoc comparisons for dopamine and glutamate, and a Kruskal-Wallis with Dunn’s posthoc comparisons for acetylcholine, histamine, serotonin, adenosine, GABA, and pyrilamine. Statistical outliers were identified by Grubb’s iterative test. Although data for some compounds was transformed for analysis, only untransformed data is presented in the figures to facilitate interpretation.

## Results

**Figure 3.**
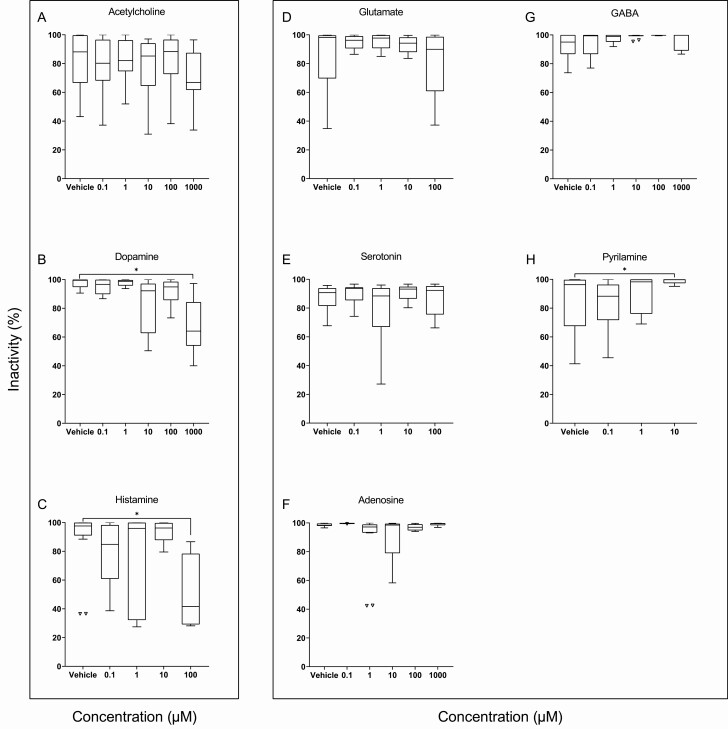
Percent of inactivity over 30 minutes per concentration of: (A) acetylcholine, (B) dopamine, and (C) histamine—all expected to decrease inactivity; and (D) glutamate, (E) serotonin, (F) adenosine, (G) GABA, and (H) pyrilamine—all predicted to increase inactivity. Asterisks denote significance (*p* < .05). The figure was plotted using the Tukey method by which the bottom and top edge of each box reflect first and third quartiles, respectively; the band inside the box is the median; the whiskers reflect minimum and maximum values, datapoints are statistical outliers not identified in Grubb’s iterative test.

**Figure 4.**
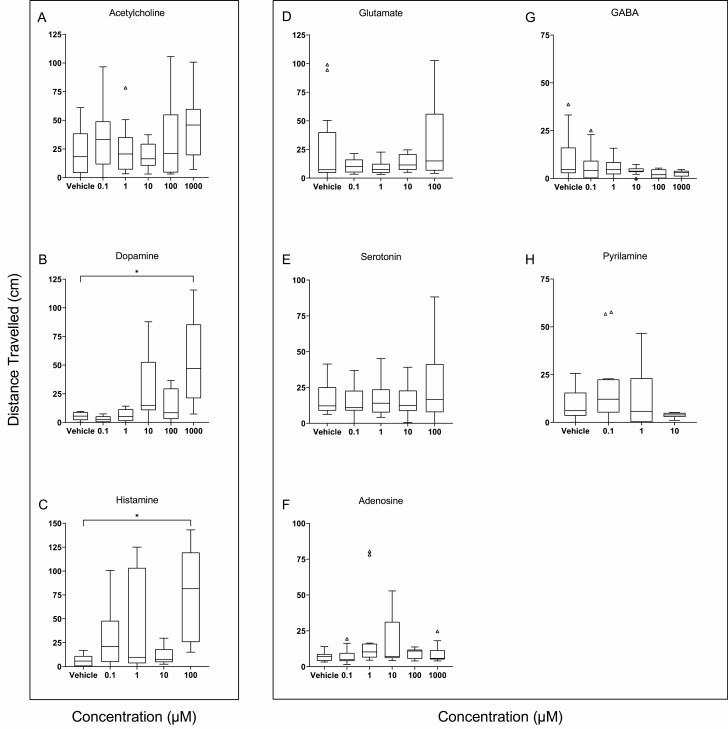
Distance traveled (cm) over 30 minutes per concentration of: (A) acetylcholine, (B) dopamine, and (C) histamine—all expected to increase distance traveled; and (D) glutamate, (E) serotonin, (F) adenosine, (G) GABA, and (H) pyrilamine—all predicted to decrease distance traveled. Asterisks denote significance (*p* < .05). Tukey’s method was employed whereby the bottom and top edge of each box reflect first and third quartiles, respectively; the band inside the box is the median; the whiskers reflect minimum and maximum values, datapoints are statistical outliers not identified in Grubb’s iterative test.

Two neurotransmitters caused the flatworms to move in a dose-dependent manner. While acetylcholine did not reduce the amount of inactivity (F_5,76_ = 0.660, *p* = .656, Figure 3A), both dopamine (H = 20.880, df = 5, *p* < .001, Figure 3B) and histamine (H = 11.852, df = 4, *p* = .018, Figure 3C) decreased inactivity, notably at the highest concentrations tested. When the behavioral response was quantified as distance traveled, again dopamine (F_5,41_ = 7.009, *p* < .001, Figure 4B) and histamine (H = 14.550, df = 4, *p* = .012, Figure 4C), but not acetylcholine (F_5,70_ = 1.368, *p* = .247, Figure 4A), showed increased movement. The absence of an effect of acetylcholine could be due to greater variance observed in the control group.

Of the five compounds expected to induce restfulness, results were neurotransmitter-specific. Glutamate (H = 4.516, df = 4, *p* = .341, Figure 3D) and serotonin (H = 3.347, df = 4, *p* = .482, Figure 3E) had no effect on inactivity. Adenosine showed an overall effect (H = 17.75, df = 5, *p* = .003, Figure 3F), but no significant pairwise differences. GABA displayed a nonsignificant trend to increase restfulness in a dose-dependent manner (H = 10.40, df = 5, *p* = .065, Figure 3G). Pyrilamine also increased inactivity (H = 7.708, df = 3, *p* = .052, Figure 3H). Effects of these four neurotransmitters were less apparent when looking at the distance traveled: glutamate (F_5,63_ = 0.988, *p* = .433, Figure 4D), serotonin (H = 1.094, df = 4, *p* = .895, Figure 4E), and adenosine (H = 9.976, df = 5, *p* = .076, Figure 4F) had no effect on movement. The dose-dependent mean decline in distance traveled under GABA was nonsignificant (H = 7.581, df = 5, *p* = .181, Figure 4G). Although pyrilamine had increased inactivity, it did not reduce how far the flatworms moved (F_3,42_ = 1.577, *p* = .209, Figure 4H).

## Discussion

**Figure 5.**
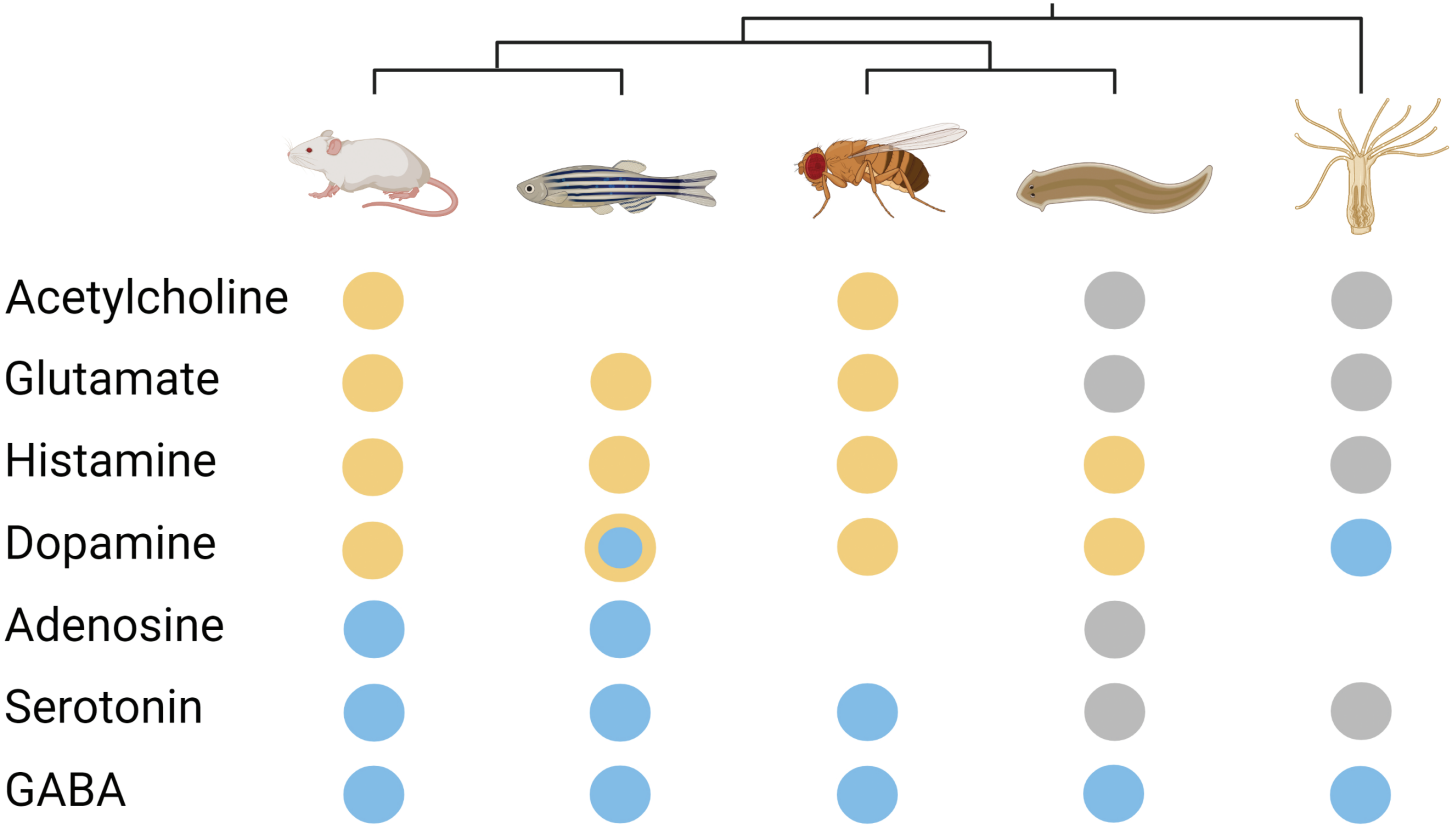
Summary of the effects of wake- (yellow) and sleep- (blue) promoting neurotransmitters across diverse animals that include mice, zebrafish, fruit flies, flatworms, and *Hydra*. Colored circles indicate behavioral responses; gray circles denote the absence of a behavioral response; and the absence of a circle itself means that compound has yet to be tested for that taxon and/or that the receptor has not been identified. The blue-within-yellow circle for dopamine-exposed zebrafish reflects the added complexity that receptor type matters for the behavioral output. Along the top of the panel is a phylogenetic tree showing evolutionary relatedness among the five species. Simple (*Hydra*) and secondarily simplified (flatworms) animals have fewer active neurotransmitters than more complex animals (mice, zebrafish, fruit flies). Only GABA maintains a somnogenic effect across all taxa studied. Created with BioRender.com. For Color, please refer the online images.

Evolutionarily conserved neurotransmitter systems regulate sleep and wakefulness in animals (Figure 5). Briefly, dopamine and histamine promote wakefulness in mammals [14] and fruit flies [35,36], and stimulate movement in flatworms. Thus, the increased distance traveled, and decreased inactivity, observed in flatworms exposed to dopamine and histamine likely reflects an increase in wakefulness. Owing to this stimulating effect of histamine, it is not surprising that the histamine antagonist pyrilamine, which is broadly sleep-promoting in other taxa [10,37,38], increased inactivity—and presumably sleep—in flatworms. The only neurotransmitter hinted to induce inactivity in flatworms was GABA. GABA stimulates sleep in all species studied from mammals and other vertebrates to the simplest invertebrates [11,14,15,25,39], suggesting that the mean increase in inactivity we observed in GABA-treated flatworms may reflect an increase in sleep. The great evolutionary longevity of GABA as a somnogenic neurotransmitter suggests that GABA may have been the first neurotransmitter to regulate sleep in animals, whose sleep-promoting effect has been retained over evolutionary time. Acetylcholine did not elicit wakefulness, nor did glutamate, serotonin, or adenosine elicit sleep in flatworms. The congruity of these findings with similar observations in *Hydra* [11], suggest that these neurotransmitters attained their state-regulating roles only with the appearance of more complex invertebrates (e.g., ecdysozoans and perhaps nonplatyhelminth lophotrochozoans) and vertebrates (see below). Overall, it appears that more complex animals (mice, zebrafish) have a greater complement of sleep- and wake-regulating neurotransmitters than simple (*Hydra*) and secondarily simplified (flatworms) animals.

### Evolutionary position of flatworms

Before casting our results into the broader literature further, it is important to understand the phylogenetic position of flatworms and their evolutionary relationship with other groups of animals. Historically, platyhelminth flatworms were considered to be the earliest branching lineage of bilaterally symmetric animals [40]. This conclusion was based (in part) on the observation that these simple animals lack circulatory, respiratory, and endocrine systems found in other bilaterally symmetric animals [41]. However, more recent studies have challenged this view and place the platyhelminths as the sister to annelids and mollusks in the superphylum Lophotrochozoa [42,43]. Sister to the Lophotrochozoa is another superphylum called Ecdysozoa (or molting animals) that notably includes arthropods and nematodes. Under this phylogeny of invertebrates, the simplified physiology of flatworms is now thought to reflect evolutionary losses rather than the retention of a “primitive” basal condition (Figure 5). This secondary simplification makes the behavioral and biochemical patterns identified here even more salient. For instance, the retention of GABA as the sole neurotransmitter regulating sleep in flatworms, suggests a fundamental functional importance.

### Wake-promoting neurotransmitters in flatworms

Dopamine is a (largely) wake-promoting neurotransmitter found across diverse animals, notably mammals and fruit flies [44] (Figure 5). The behavioral response to dopamine is more complex in zebrafish. Using a variety of dopamine D1-receptor agonists, Rihel et al. [17] found evidence for a sedation effect of dopamine in zebrafish, while D1 agonists tested on mammals are arousing. Given these complexities, understanding the evolutionary relatedness between the eight zebrafish dopamine receptors to the five mammalian receptors would provide added insight into how dopamine regulates sleep and/or wakefulness in these vertebrates. Dopamine appears to induce sleep in *Hydra* [11]; however, which dopamine receptors mediate the effect, and any homology with dopamine receptors in vertebrates, is unknown. Dopamine promotes wakefulness in flatworms. This observation agrees with work demonstrating the presence of dopamine receptors in the central nervous system of flatworms [27,45], whose distribution overlaps with that of serotonergic neurons [20,25]. Taken together, dopamine appears to be wake-promoting in flatworms, fruit flies, variably in zebrafish, and in mammals.

Histamine has been found in the central nervous system of mammals and zebrafish [38] where it plays a role in regulating several body functions, including wakefulness [46]. Histamine is also present in the nervous system of invertebrates, including cockroaches, fruit flies, locusts, and (platyhelminth) cestodes [47–50], and in the photoreceptor cells of the flatworm eyespots (ocelli) [20]. Among invertebrates, studies on the biochemical regulation of sleep and wakefulness have shown that histamine keeps flies and flatworms awake [24,25]. Conversely, the absence of a stimulating effect on *Hydra* might suggest histamine adopted a wake-promoting role only after the appearance of bilaterally symmetric animals (Figure 5).

### Sleep-promoting compounds in flatworms

Pyrilamine is a H1 histamine receptor antagonist. Given the wake-promoting effects of histamine, perhaps it is unsurprising to find widespread sleep-promoting effects of pyrilamine across diverse and distantly related animals. Pyrilamine induces sleep in zebrafish [38] and mammals, at least at some dosages [37,46]. The response of fruit flies to pyrilamine has yet to be studied, but the upside-down jellyfish (*Cassiopeia* spp.) pulsed with a longer, more variable, inter-pulse interval indicative of sleep when exposed to pyrilamine [10]. Similarly, flatworms showed an increase in restfulness when administered pyrilamine, providing additional evidence that histamine is wake-promoting.

GABA is expressed throughout the mammalian brain and is one of the main neurotransmitters regulating sleep via GABAergic neurons [51]. GABA is thought to be of major importance to flatworms as well owing to high endogenous concentrations and widespread distribution of receptors throughout the nervous system [19]. More broadly, GABA is the only neurotransmitter found to have a consistent, somnogenic effect across all animals studied, including vertebrates [32,37] and diverse and distantly related invertebrates [6,11]. Thus, GABA appears to have held an evolutionarily conserved role in the biochemical regulation of sleep (Figure 5). Interestingly, Keenan et al. [52] investigated the effect of GABA on electrical activity in the nerve cords of another flatworm, *Notoplana acticola*. Using evoked responses, GABA reversibly suppressed neuronal activity. This GABA-mediated neuronal silence might reflect GABA-induced sleep, given other invertebrates show reduced neuronal activity during sleep, including honey bees (*Apis mellifera*) [53] and fruit flies [54,55]. Therefore, GABA-mediated neuronal quiescence and the mean increase in restfulness we observed in GABA-treated flatworms, suggests that GABA is involved in regulating sleep in flatworms. Nonetheless, it is important to verify this conclusion using other measures of brain activity, such as local field potentials or calcium imaging, in flatworms [20].

### Neurotransmitters that did not yield a behavioral response in flatworms

Acetylcholine was expected to promote wakefulness in flatworms, as it does in mammals [14] and fruit flies [55], but surprisingly did not influence either the distance traveled by flatworms or the amount of inactivity. Acetylcholine has been found within the nervous system of flatworms where it can trigger either muscle contraction or relaxation in a dose-dependent manner in both parasitic and free-living flatworms [24,25,27,29,30]. The absence of a movement-inducing effect here could be artifactual, owing to unexplained increased variance in the control group for this neurotransmitter. This increased vehicle variance cannot be explained by the time-of-day, as dopamine and histamine show much more conserved vehicles; nor by time-of-year, because acetylcholine data was collected at the time as other compounds that showed unremarkable variation in their control. Consequently, there is value in investigating the role of acetylcholine further in the lives of flatworms.

When we designed this study, we thought that glutamate should induce sleep in flatworms, based on an ostensibly sleep-promoting role in fruit flies [15,34]. As such, we exposed flatworms to glutamate during the night when they would otherwise be awake. However, we since read that glutamate is wake-active in vertebrates and fruit flies alike [56]. While glutamate has been shown to trigger muscular contractions in flatworms [20,24,25], there is no evidence of increased or decreased activity during the waking period in the current study. The results found here are similar to those seen in *Hydra* where no effect of glutamate was found regardless of photoperiod [11].

In vertebrates, there are several adenosine receptor subtypes involved in inducing sleep [12,57–62]. *Drosophila* pose a contrast here, where the *Drosophila* homolog of the mammalian A_2A_ receptor (responsible for sleep in mammals), *dAdoR,* does not regulate sleep-wake behaviors [58]. Not only did flatworms not respond to adenosine, but caffeine (an adenosine antagonist) has no effect on the locomotion of flatworms [63]. There have been no studies looking at the effects of adenosine in cnidarians.

Although serotonin promotes sleep in vertebrates and fruit flies [16,64,65], flatworms and *Hydra* show no response to serotonin. Despite the absence of an effect in flatworms, serotonin is found throughout the central- and peripheral nervous system in platyhelminths [19,21,22]. Itoh & Igarashi [21] showed that circulating levels of serotonin are lower during the day (when flatworms are predominantly asleep [9]), than during the night. In the current study, there was no evidence that serotonin elicited either an increase or decrease in activity, perhaps concurring with the hypothesis that serotonin is mainly involved in timekeeping and regeneration [21]. While other studies demonstrate that serotonin can increase and decrease locomotion depending on the concentration, no tests have been conducted in the context of circadian rhythms [23–25].

To study sleep, particularly in invertebrates, looking at the whole animal and specific behaviors is important [11,66]. A limitation of this approach relates to the interpretation of an absence of a response. And so, it is equally important to understand the distribution, and types, of receptors, and their homologies across species. Notwithstanding, GABA, dopamine, and histamine have been important neurochemical messengers since (at least) the appearance of the centralized nervous system. The conserved function of these neurotransmitters regulating sleep/wake states shows promise for further advances in sleep research.

## Supplementary Material

zsac053_suppl_Supplementary_Table_S1
