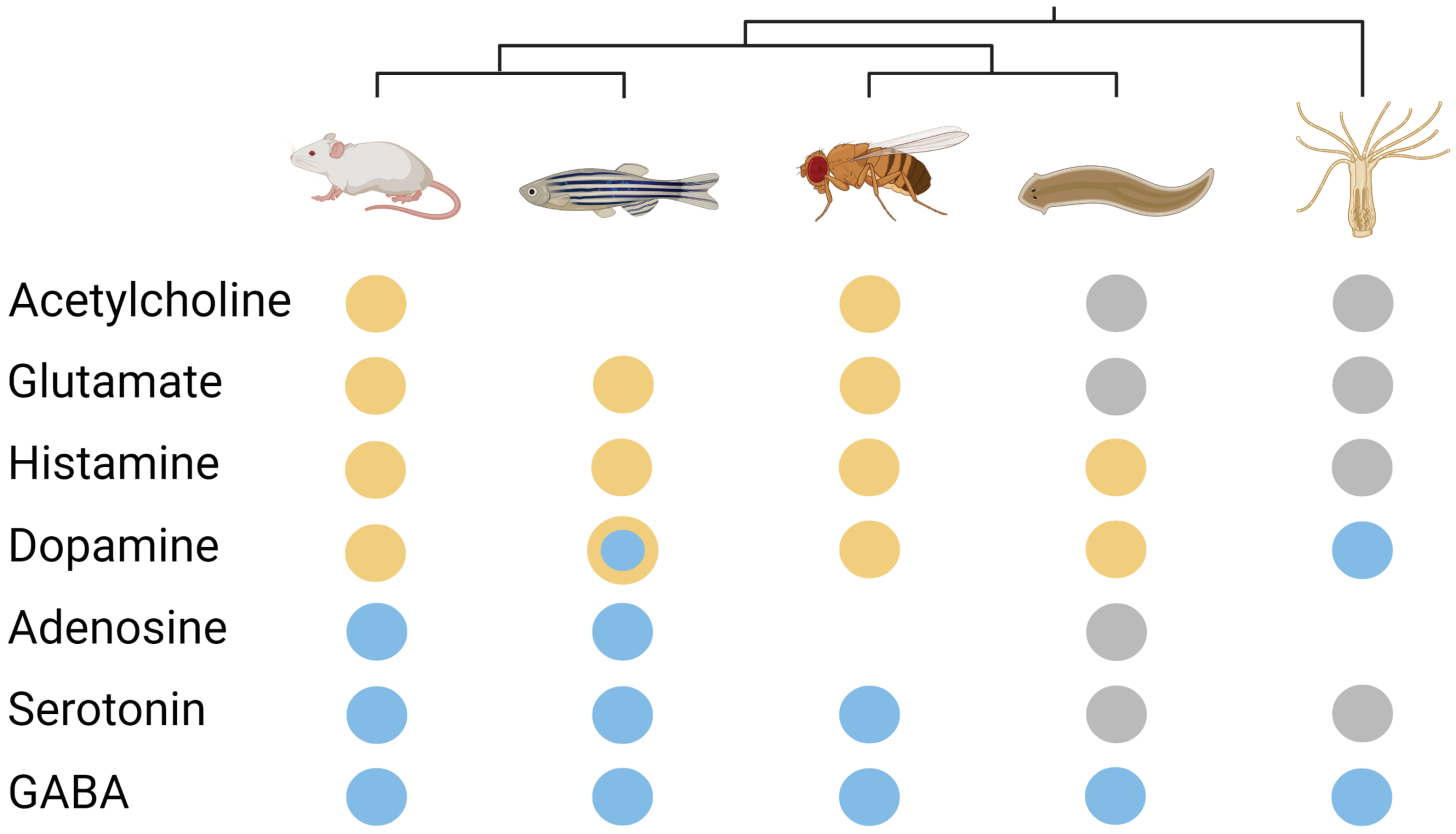# Correction to: Neurotransmitters of sleep and wakefulness in flatworms

**DOI:** 10.1093/sleep/zsac127

**Published:** 2022-06-06

In the originally published version of this manuscript, an incorrect image was used for Figure 5 in error. The ‘GABA’ circle for flatworms should be blue. This error has been corrected.