# Supplementary material for: Neurotransmitters of sleep and wakefulness in flatworms
Source: Sleep. 2022 Mar 7;45(5):zsac053. doi: 10.1093/sleep/zsac053 (PMC9216492; doi:10.1093/sleep/zsac053)
Supplement: zsac053_suppl_Supplementary_Table_S1 [file zsac053_suppl_supplementary_table_s1.docx]

**Neurotransmitters of sleep and wakefulness in flatworms**

Shauni E. T. Omond^1*^, Matthew W. Hale^2^, and John A. Lesku^1*^

^1^School of Agriculture, Biomedicine and Environment, La Trobe University, Melbourne, Australia

^2^School of Psychology and Public Health, La Trobe University, Melbourne, Australia

^*^Correspondence: shauni.omond@gmail.com; j.lesku@latrobe.edu.au

Table S1. Experimental sample sizes. Sample size per concentration per compound for distance travelled / percent inactivity (modest differences arose from the Grubb’s iterative test for removing statistical outliers). A dash in the column indicates that no samples were analyzed as either the concentration produced an adverse reaction, oxidized too quickly, or had a low number not conducive to meaningful statistical analysis.

|  | Vehicle | 0.1 μM | 1 μM | 10 μM | 100 μM | 1000 μM |
| --- | --- | --- | --- | --- | --- | --- |
| Acetylcholine | 11/12 | 15/15 | 12/13 | 10/13 | 15/15 | 13/14 |
| Dopamine | 06/06 | 07/07 | 06/06 | 07/07 | 10/10 | 11/11 |
| Histamine | 12/14 | 13/13 | 14/14 | 12/12 | 07/07 | – |
| Glutamate | 16/16 | 16/16 | 11/11 | 12/12 | 13/13 | – |
| Serotonin | 16/16 | 16/16 | 11/13 | 12/11 | 14/13 | – |
| Adenosine | 10/09 | 14/10 | 13/13 | 10/10 | 09/09 | 10/08 |
| GABA | 15/15 | 15/15 | 14/13 | 11/11 | 10/09 | 09/11 |
| Pyrilamine | 12/14 | 12/12 | 14/14 | 08/08 | – | – |
